# *Saccharomyces boulardii* CNCM I-745 supplementation during and after antibiotic treatment positively influences the bacterial gut microbiota

**DOI:** 10.3389/fmed.2023.1087715

**Published:** 2023-08-04

**Authors:** Madeleine Spatz, Yazhou Wang, Alexia Lapiere, Gregory Da Costa, Chloé Michaudel, Camille Danne, Marie-Laure Michel, Philippe Langella, Harry Sokol, Mathias L. Richard

**Affiliations:** ^1^INRAE, AgroParisTech, Micalis Institute, Université Paris-Saclay, Jouy-en-Josas, France; ^2^Paris Center for Microbiome Medicine (PaCeMM), Fédération Hospitalo-Universitaire, Paris, France; ^3^INSERM UMRS-938, Centre de Recherche Saint-Antoine, CRSA, AP-HP, Sorbonne Université, Paris, France

**Keywords:** microbiota, mycobiota, antibiotics, *Saccharomyces boulardii* CNCM I-745, treatment

## Abstract

**Introduction:**

Antibiotic effects on gut bacteria have been widely studied, but very little is known about the consequences of such treatments on the mycobiota, the fungal part of the microbiota and how the length of administration influences both microbiota. Here, we examined the effect of antibiotics (ATB) on the composition of bacterial and fungal microbiota and how the administration of *Saccharomyces boulardii* CNCM I-745 influences both microbiota.

**Methods:**

In order to get closer to the human microbiota, the mice used in this study were subjected to fecal microbiota transfer (FMT) using human feces and subsequently called human microbiotaassociated (HMA) mice. These mice were then treated with amoxicillinclavulanate antibiotics and supplemented with *S. boulardii* during and after ATB treatment to understand the effect of the yeast probiotic on both bacterial and fungal microbiota. Bacterial and fungal microbiota analyses were done using 16S and ITS2 rRNA amplicon-based sequencing.

**Results:**

We showed that the administration of *S. boulardii* during ATB treatment had very limited effect on the fungal populations on the long term, once the yeast probiotic has been cleared from the gut. Concerning bacterial microbiota, *S. boulardii* administration allowed a better recovery of bacterial populations after the end of the ATB treatment period. Additionally, 16S and ITS2 rRNA sequence analysis revealed that 7 additional days of *S. boulardii* administration (17 days in total) enhanced the return of the initial bacterial equilibrium.

**Discussion:**

In this study, we provide a comprehensive analysis of how probiotic yeast administration can influence the fungal and bacterial microbiota in a model of broad-spectrum antibiotherapy.

## Background

The gut microbiota has been widely studied for the last two decades, and the development of next-generation sequencing (NGS) has allowed the identification of its pivotal role in various pathologies in recent years. Indeed, the gut microbiota has been identified as a cofactor in diseases of the digestive tract, such as inflammatory bowel disease (IBD) and colorectal cancer (CRC), as well as in diseases indirectly related to the gut, such as allergies, diabetes, obesity and liver diseases (1–8).

Probiotics are living, active organisms that have beneficial effects on the health of the host and can be found in fermented foods, dietary supplements, and even drugs (9). They can be used to improve gastrointestinal disorders, such as diarrhea (infectious and antibiotic-associated), necrotizing enterocolitis, *Clostridioides difficile* infection, IBD (ulcerative colitis and Crohn’s disease) and irritable bowel syndrome (IBS) (10, 11). There are several probiotic products, most of which belong to the group of lactic acid-producing bacteria, but very few are yeasts. The few that are available belong to the *Saccharomyces* genus (e.g., *S. cerevisiae* and *S. boulardii*). However, *S. boulardii* CNCM I-745 was the first one to be discovered and is widely used throughout the world. Discovered a century ago in 1920, *S. boulardii* has been shown to prevent and reduce the occurrence of diarrhea associated with antibiotic treatment (12–14). It also exerts protective properties in the treatment of *Clostridioides difficile* and *Helicobacter* infections (15–17). It has also been identified as protective in mouse models of IBD and IBS (18, 19).

Antibiotics are used in humans to treat a large number of infections with rather high efficacy. However, they also disrupt intestinal microorganisms, and dysbiosis can be detected long after the end of antibiotic treatment (20). The effects of antibiotics on the bacterial microbiota are particularly well described, but very little is known about their impact on the fungal microbiota (mycobiota).

Kabbani and colleagues studied the effect of the combination of *Saccharomyces boulardii* CNCM I-745 and amoxicillin-clavulanate on the gut microbiota in humans and reported a milder effect on microbiota shifts in the presence of *S. boulardii*, including less overgrowth of *Escherichia* and a reduction in antibiotic-associated diarrhea (13). However, this study, like the majority of research on the gut microbiota, focused solely on the bacterial component, while other members of the gut microbiota, such as fungi, viruses and archaea, were neglected.

The objectives of this study were to evaluate the effects of an antibiotic (amoxicillin-clavulanate – ATB) and the probiotic *Saccharomyces boulardii* CNCM I-745 on the gut fungal and bacterial microbiota in HMA mice. We chose the amoxicillin-clavulanate since it is one of the most commonly used antibiotics in the primary care setting (21). We also examined the impact of *S. boulardii* administration after the end of ATB treatment. *S. boulardii* was able to attenuate the dysbiosis associated with ATB when administered during treatment and maintain a higher diversity when administered continuously during and after ATB treatment.

Therefore, this study will help us to understand the effect of *S. boulardii* CNCM I-745 administration during ATB treatment on both bacterial and fungal microbiota to improve medical practices in the future.

## Materials and methods

### Mice

**Figure 1:**
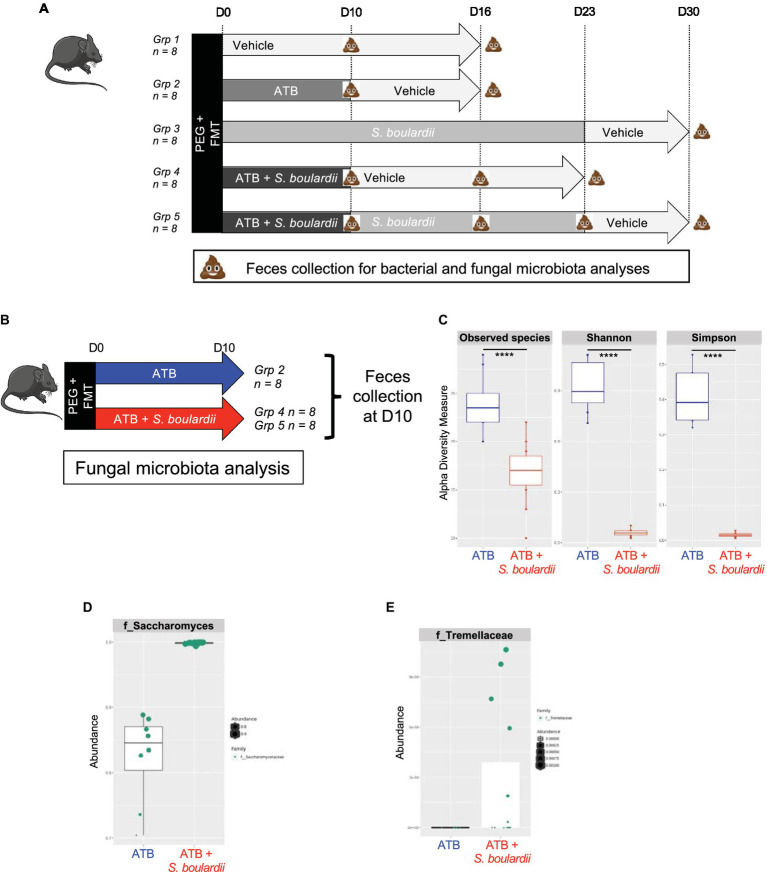
Effect of *Saccharomyces boulardii* supplementation during antibiotic treatment on the mycobiota. **(A)** Setup of the entire experiment to decipher the effect of the yeast probiotic *S. boulardii* on the fungal and bacterial microbiota after antibiotic treatment (ATB). Group (Grp) 1: HMA mice treated with the Vehicle for 16 days, *n* = 8; Grp 2: HMA mice treated with the ATB for 10 days, then Vehicle for the following 6 days, *n* = 8; Grp 3: HMA mice treated with *S. boulardii* for 23 days, then Vehicle for the following 7 days, *n* = 8; Grp 4: HMA mice treated with the ATB and *S. boulardii* for 10 days, then Vehicle for the following 13 days, *n* = 8; and Grp 5: HMA mice treated with the ATB and *S. boulardii* for 10 days, then *S. boulardii* for the following 13 days, then Vehicle for the following 7 days, *n* = 8. **(B–E)** Human microbiota-associated (HMA) mice treated with the ATB or ATB plus *S. boulardii* for 10 days: Grp 2 “ATB” *n* = 8; Grp 4 and 5 “ATB + *S. boulardii*” *n* = 16 (Grp 4 *n* = 8, Grp 5 *n* = 8), the experiment was performed 2 times. **(B)** Experimental design for the administration of the ATB and probiotic over 10 days. **(C)** α–Diversity of the fungal microbiota (ITS2 rRNA) in the fecal microbiota, described by observed species and Shannon and Simpson indexes. **(D,E)** Taxa selected with the differential abundance analysis based on the negative binomial distribution (DESeq2) with padj <0.05.

Six-week-old female C57BL/6 J mice were purchased from Janvier Laboratory (Le Genest, France) and used 1 week after delivery. Animals were kept in humidity- and temperature-controlled rooms under a 12 h light–dark cycle and had access to a chow diet and water *ad libitum*. Mice were grouped by types of treatment in cages with a maximum of 4 mice per cages as follow (Figure 1):

Group (Grp) 1: HMA mice treated with the Vehicle for 16 days, *n* = 8; Grp 2: HMA mice treated with the ATB for 10 days, then Vehicle for the following 6 days, *n* = 8; Grp 3: HMA mice treated with *S. boulardii* for 23 days, then Vehicle for the following 7 days, *n* = 8; Grp 4: HMA mice treated with the ATB and *S. boulardii* for 10 days, then Vehicle for the following 13 days, *n* = 8; and Grp 5: HMA mice treated with the ATB and *S. boulardii* for 10 days, then *S. boulardii* for the following 13 days, then Vehicle for the following 7 days, *n* = 8. Depending on the question the feces from each group were collected at different time points identified in each figure. As Grp 4 and 5 received the exact same treatment till day 10 (ATB plus *S. boulardii*), the 4 cages of Grp 4 and 5 were identical for our analysis till day 10. Considering this, we decided to increase the size of the group treated with ATB and *S. boulardii* at day 10 from 8 to 16 by using all mice from Grp 4 and Grp 5 collected in all 4 cages.

Every experiment was repeated two times, so a total of 40 mice were used in the results presented in this study but 80 were used for the repetition of the experiments. All experiments were performed in accordance with the ethics committee “Comité d’Ethique en Experimentation Animale” (COMETHEA C2EA – 45, Jouy en Josas, France).

### Fecal microbiota transfer

Mice received feces from a healthy donor as previously described (22). Briefly, feces from humans were collected and immediately stored at 4°C in an anaerobiosis generator (Genbox, Biomérieux, Capronne, France) to favor the preservation of anaerobic bacteria. Samples were processed within 24 h in a Coy chamber. The feces were rapidly diluted 100-fold in brain heart infusion (BHI, Becton Dickinson, Franklin Lakes, United States) supplemented with 0.5 mg/mL L-cysteine (Sigma–Aldrich, St. Louis, MO, United States) and 20% skim milk (Becton Dickinson, Franklin Lakes, United States) (vol/vol) and stored in aliquots at −80°C. This ready-to-use fecal suspension was used for FMT to the mice.

Mice were fasted for 1 h and then subjected to bowel cleansing by oral-gastric gavage with PEG (polyethylene glycol, Macrogol 4,000, Fortrans, Ipsen Pharma, France). Bowel cleansing with PEG was performed only once. 4 hours later, mice received human feces by oral gastric gavage (350 μL of resuspended feces prepared as described above). Mice were then allowed free access to food and water. FMT was repeated once a week for 3 weeks before the antibiotic was given by oral gavage without any PEG treatment.

### Antibiotic treatments and gavage with *Saccharomyces boulardii* CNCM I-745

Amoxicillin-clavulanate (5,1, 150 mg/kg, Sandoz, Bale, Switzerland) was resuspended in NaCl and administered to mice daily by intragastric gavage for 10 days. Concentration of the mice ATB treatment was determined using the quantity per kilogram prescribed to human.

*Saccharomyces boulardii* CNCM I-745 (Biocodex, Gentilly, France) was used in this study. Lyophilized yeast was resuspended in Vehicle (60 mg/day/mouse) and directly administered by 200 μL intragastric gavage during and after the ATB treatment. The control group was administered 200 μL of Vehicle daily.

### Fecal samples collection

Fecal samples were collected in the morning and frozen for microbiota analysis. Mice were individually placed in a sterile container to defecate and fecal pellets were collected using sterile tips and placed in a sterile 1.5 mL tube for immediate freezing in dry ice. All samples were stored at −80°C until use.

### Fecal DNA extraction

Fecal total DNA was extracted from weighed content samples as previously described, with modifications (23). After nucleic acid precipitation with isopropanol, DNA suspensions were incubated overnight at 4°C and centrifuged at 20,000 × g for 30 min. The supernatants were transferred to a new tube containing 2 μL of RNase (RNase A, 10 mg/mL; EN0531; Fermentas, Villebon sur Yvette, France) and incubated at 37°C for 30 min. Nucleic acids were precipitated by the addition of 1 mL of absolute ethanol and 50 μL of 3 M sodium acetate and centrifuged at 20,000 × g for 10 min. The DNA pellets were washed with 70% ethanol 3 times, dried and resuspended in 100 μL of Tris-EDTA (TE) buffer (10 mM Tris–HCl, 1 mM EDTA, adjusted pH 8).

The DNA suspensions were stored at −20°C for real-time qPCR analysis of the 16S or ITS2 rRNA sequences analyses.

### 16S and ITS2 rRNA sequencing

Bacterial diversity was determined for each sample by targeting a portion of the ribosomal genes. PCR was performed to prepare amplicons using V3-V4 oligonucleotides (primers PCR1F_460: 5′-CTTTCCCTACACGACGCTCTTCCGATCTACGGRAGGCAGCAG-3′ and PCR1R_460: 5′-GGAGTTCAGACGTGTGCTCTTCCGATCTTACCAGGGTATCTAAT CCT-3′) (24–26). Amplicon quality was verified by gel electrophoresis, and samples were sent to the @BRIDGe platform for sequencing on an Illumina MiSeq (Illumina, San Diego, CA, United States).

A similar approach was used for fungal microbiota using the primers ITS2 (sense) 5′-GTGARTCATCGAATCTTT-3′ and (antisense) 5′-GATATGCTTAAGTTCAGCGGGT-3′ and the optimized and standardized ITS2 rRNA-amplicon-library preparation protocol (Metabiote, GenoScreen, Lille, France).

### 16S and ITS2 rRNA sequence analysis

The 16S rRNA sequences were demultiplexed and quality filtered using the QIIME version 2.1.0 software package (27). The sequences were then assigned to ASVs (amplicon sequence variant) using the DADA2 algorithm (28) with a 97% pairwise identity threshold and classified taxonomically using the SILVA reference database (version 13.8) for bacteria (29). For the ITS2 rRNA sequences, data were processed using the FROGS pipeline (30) for sequence quality control, and filtering and affiliation of taxa was performed with the UNITE ITS database (version 8_2) (31) using the FROGS guidelines for ITS data.^1^ Rarefaction analysis was performed and used to compare the relative abundance of OTUs across samples. α-diversity was estimated using the Shannon diversity index or the number of observed species. β-diversity was measured using the Jaccard distance matrix and was used to build principal coordinates analysis (PCoA) plots. Using these tools, we did not observe any cage effect in our different sets of experiments. Differential abundance analysis based on the negative binomial distribution (DESeq2) algorithm was used to identify taxa that were specific to the treatment (32). The raw sequence data were deposited in the SRA database from the NCBI under the following accession numbers (PRJNA896895).

### Statistical analysis

GraphPad Prism version 7 (San Diego, CA, United States) was used to perform all analyses and prepare graphs. For all data displayed in graphs, the results are expressed as the mean ± SEM (*n* = 7 to 12 per group). For comparisons between two groups, Student’s *t* test for unpaired data was used. For comparisons among more than two groups, one-way analysis of variance (ANOVA) and a *post hoc* Tukey or Dunnett test were used. For all statistical tests, differences with a *p* value less than 0.05 were considered statistically significant: *p < 0.05, **p < 0.01, ***p < 0.001. Statistical significance of sample grouping for β-diversity analysis was performed using Permanova method (999 permutations).

## Results

The evaluation of the impact of the yeast probiotic on the fungal and bacterial microbiota after ATB treatment was assessed using a complex setup of *in vivo* experiments. The complete setup is presented in Figure 1A with 5 different groups of 8 mice each. However, for analysis purposes we did not analyze all timepoints but we choose to focus on specific time points in order to decipher the impact of each treatment on both fungal and bacterial microbiota. At these specific time points feces from mice of the selected treated groups were collected and the microbiota composition analyzed. The fungal and bacterial microbiota analyses are separately presented as they were impacted in different manners.

### *Saccharomyces boulardii* CNCM I-745 administration do not affect the fungal population on the long term

As the first step of characterization of the yeast probiotic effects on the mycobiota on ATB treated mice, we evaluated the effect of *Saccharomyces boulardii* CNCM I-745 administration on the mycobiota of mice treated for 10 days with ATB by sequencing the ITS2 region of DNA from fecal samples (Figure 1B). We chose to study HMA mice to help further understand fungal and bacterial interactions within the human gut microbiota. Daily administration of yeast biased the study of α- or β-diversity because the constant and very high levels of *S. boulardii* skewed the ITS2 rRNA analyses and potentially masked the modifications of other taxa. As expected, administration of *S. boulardii* during ATB treatment increased the levels of the *Saccharomycetaceae* family and reduced the α-diversity of the fungal community (Figure 1C) because only one species was in high abundance (Figure 1D). Using the DESeq2 R package for differential analysis, we identified 6 families that were significantly impacted by *S. boulardii:* (*p* value adjusted <0.05): *Atheliaceae*, *Debaryomycetaceae*, *Erysiphaceae*, *Hypocreales*, *Pleosporaceae* and *Tremellaceae*. However, while the higher relative abundance cannot be contested for *Tremellaceae*, the reduced relative abundance could be due only to the over-representation of *S. boulardii* in the total sequences. Additional quantitative real time PCR or CFU would be necessary for the absolute abundance characterization in order to account for actual decrease of fungi families. Nevertheless, *S. boulardii* slightly increased the levels of the *Tremellaceae* family (Figure 1E) and seemed to contained the development of five specific families that would need further investigation using absolute quantification.

In an attempt to see the effect of *S. boulardii* administration without the cofounding effect of the gavage with *S. boulardii* we compared the fungal microbiota of mice under antibiotic treatment with or without supplementation with *S. boulardii* but 1 week after the discontinuation of the *S. boulardii* administration (Supplementary Figure S1A). In this context, α-and β-diversity analyses did not show any statistical differences as well as the specific analysis using DesEQ2 statistical tools (Supplementary Figure S1B) suggesting no long-term effect of the yeast probiotic supplementation on the fungal microbiota.

### One week after stopping all the treatments, the mycobiota returned to equilibrium

**Figure 2:**
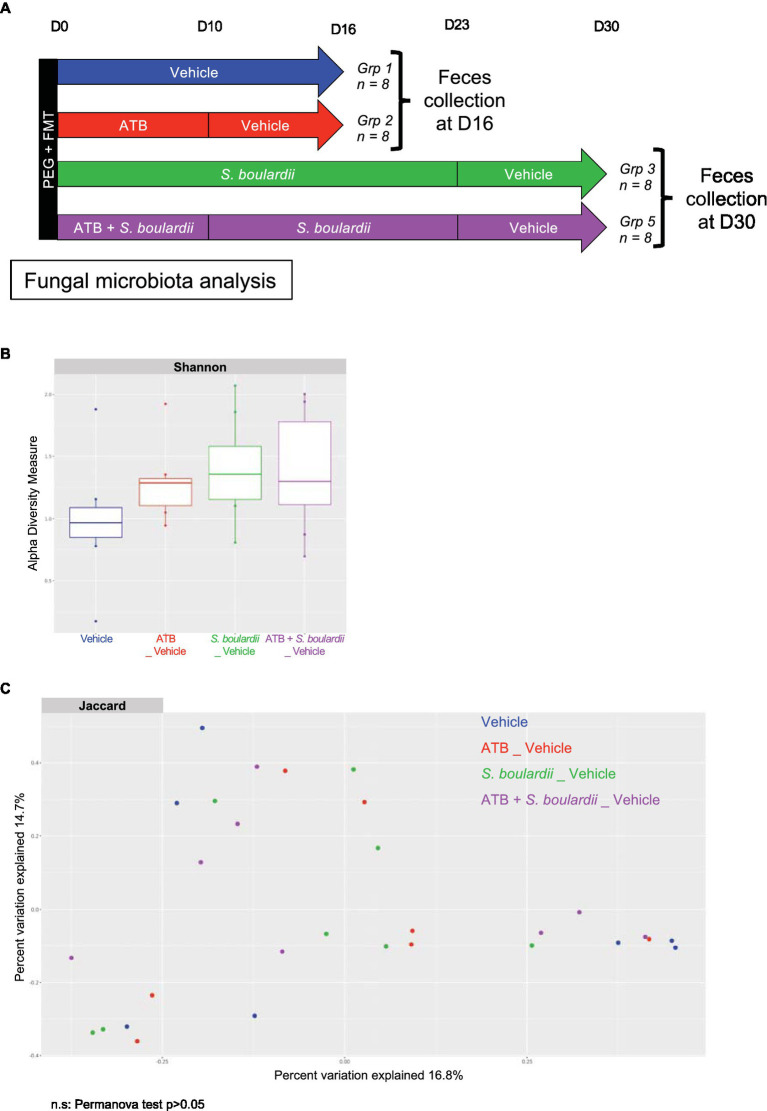
One week after stopping all the treatments, the mycobiota returned to equilibrium. **(A–C)** HMA mice treated (ATB _ Vehicle) or not (Vehicle) with the ATB for 10 days and *S. boulardii* for 30 days with (ATB + *S. boulardii* _ Vehicle) or without the ATB (*S. boulardii* _ Vehicle). Grp 1 “Vehicle” *n* = 8; Grp 2 “ATB _Vehicle” *n* = 8; Grp 3 “*S. boulardii* _Vehicle” *n* = 8; Grp 5 “ATB + *S. boulardii*_Vehicle” *n* = 8, the experiment was performed 2 times. **(A)** Experimental design for the administration of the ATB and probiotic over 30 days. **(B)** α–Diversity of the fungal microbiota (ITS2 rRNA) in the fecal microbiota after ATB and probiotic treatments, described by the Shannon index. **(C)** β-Diversity. Principal coordinates analysis of Jaccard distances with each sample colored according to the treatment. Groups were compared using Permanova method.

Finally, with the aim of describing the evolution of the mycobiota when the patient had stopped his medication, we examined the composition of the mycobiota 1 week after all treatments were stopped. We performed ITS rRNA sequencing of mouse feces without *S. boulardii* administration at D16, 1 week after the end of antibiotic therapy, and with *S. boulardii* administration at D30, 1 week after the end of yeast supplementation (Figure 2A). We were then able to analyze α- and β-diversities, as it is known that *S. boulardii* cannot become established in the human gut and is cleared within 24 to 48 h (33). Moreover, when administered daily to mice and then stopped, the yeast is undetectable after 1 week (34). The effect of *S. boulardii* administration 1 week after the end of supplementation showed a slight and nonsignificant increase in α-diversity (Shannon index) compared with mice that did not receive *S. boulardii* treatment (Figure 2B). An analysis of β-diversity showed no distinct clusters (Figure 2C), meaning that the mycobiota composition 1 week after the end of all treatments was similar between all groups.

### *Saccharomyces boulardii* significantly reduced the levels of several bacterial families enhanced by antibiotic treatment

While several studies have described the effects of ATB treatment and *S. boulardii* CNCM I-745 on gut bacterial microbiota composition (13), the longitudinal evaluation of *S. boulardii* administration during and after ATB treatment has rarely been reported and not in detail. Here, we followed different courses of treatment and analyzed how *S. boulardii* impacts the bacterial microbiota.

ATB treatment induced global changes in the bacterial microbiota, with a decrease in many commensal bacterial families, but these ecosystem modifications also favored the development of several other antibiotic-resistant bacteria favored by the release of many niches from sensitive bacteria. Hence, the families *Clostridia vadin BB60 group*, *Enterobacteriaceae*, *Enterococcaceae*, *Monoglobaceae, Peptostreptococcaceae* and *Tannerellaceae* were significantly induced after ATB treatment (Supplementary Figures S2A,B).

**Figure 3:**
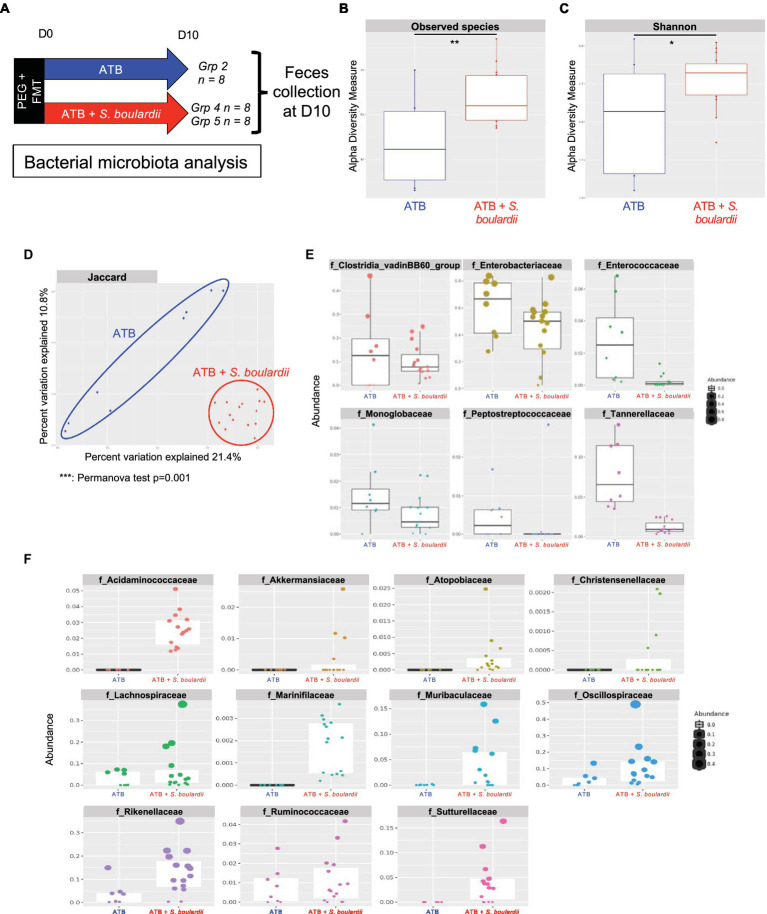
Effect of *Saccharomyces boulardii* supplementation during antibiotic treatment on the bacterial microbiota. **(A–F)** HMA mice treated with the ATB or ATB and *S. boulardii* (ATB + *S. boulardii*) for 10 days. Grp 2 “ATB” *n* = 8; Grp 4 and 5 “ATB + *S. boulardii*” *n* = 16 (Grp 4 *n* = 8, Grp 5 *n* = 8), experiment was done 2 times. **(A)** Experimental design for the administration of ATB and probiotics over 10 days. **(B,C)** α–Diversity of the bacterial microbiota (16S rRNA) in the fecal microbiota, described by **(B)** observed species and **(C)** Shannon indices. **(D)** β-Diversity. Principal coordinates analysis of Jaccard distances with each sample colored according to the treatment. Groups were compared using Permanova method. **(E,F)** Taxa selected with the differential abundance analysis based on the negative binomial distribution (DESeq2) with padj <0.05 **(E)** decreased or **(F)** increased by the probiotic treatment unless otherwise indicated (n.s.: no significant differences).

Interestingly, when *S. boulardii* was administered during ATB treatment (Figure 3A), it increased the number of observed species (Figure 3B) and the α-diversity (Shannon index) compared with mice that only received ATB treatment (Figure 3C). It also induced a global new composition of the bacterial microbiota, as illustrated by the clear clustering of the groups with or without *S. boulardii* (Jaccard index, *p* value = 0.001) (Figure 3D). Differential analysis using the DESeq2 tool showed that *S. boulardii* was able to reduce the levels of all the families increased by ATB treatment. Specifically, it reduced the levels of the 6 families significantly increased by ATB treatment: *Clostridia vadin BB60 group*, *Enterobacteriaceae*, *Enterococcaceae*, *Monoglobaceae*, *Peptostreptococcaceae* and *Tannerellaceae* (Figure 3E). *S. boulardii* was able to increase the abundances of the families *Acidaminococcaceae*, *Akkermansiaceae*, *Atopobiaceae*, *Christensenellaceae*, *Lachnospiraceae*, *Marinifilaceae*, *Muribaculaceae*, *Oscillospiraceae*, *Rikenellaceae*, *Ruminococcaceae* and *Sutterellaceae* (Figure 3F). Taken together, these data confirm a strong influence of the coadministration of *S. boulardii* CNCM I-745 during ATB treatment on the bacterial ecosystem, with the capacity to reduce the imbalance induced by ATB treatment and create an alternative bacterial ecosystem with the enhancement of other families.

### Continuous *Saccharomyces boulardii* administration for two weeks after antibiotic treatment favors the expansion of commensal bacteria but contains the development of antibiotic-resistant bacteria

**Figure 4:**
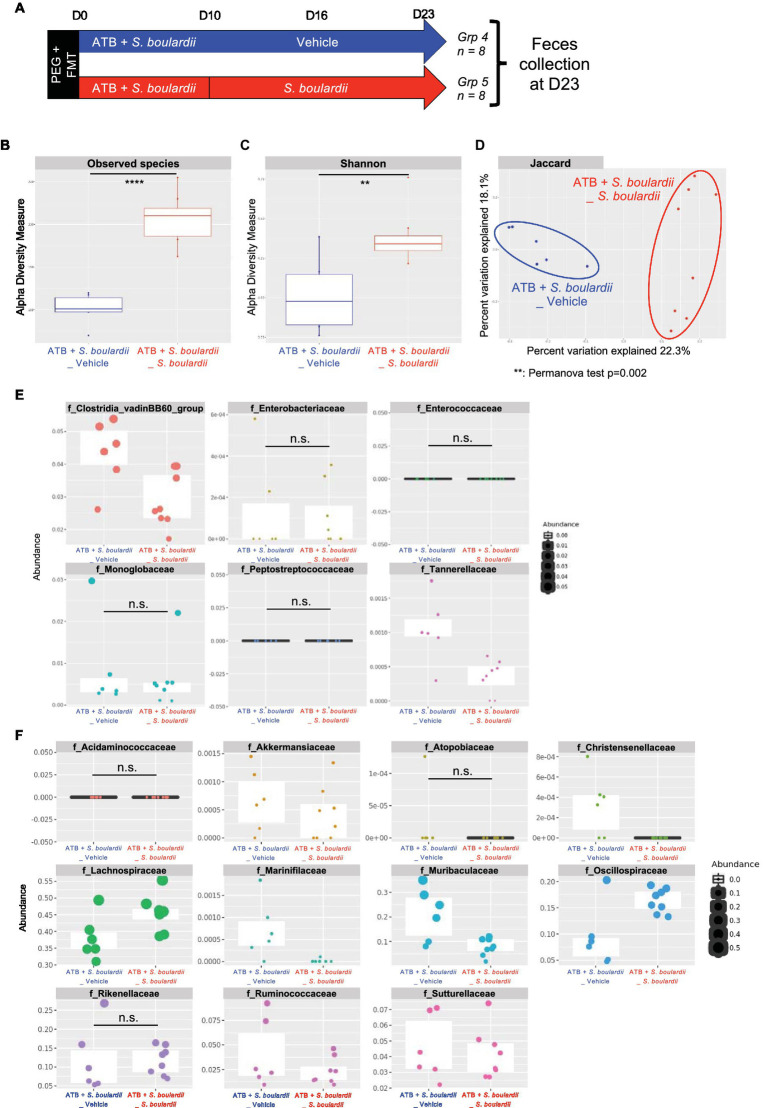
Effect of *Saccharomyces boulardii* supplementation on the bacterial microbiota for 2 weeks after antibiotic therapy. **(A–F)** HMA mice treated with the ATB and *S. boulardii* for 10 days and then no treatment for 2 weeks (ATB + *S. boulardii* _Vehicle) or two more weeks of *S. boulardii* (ATB + *S. boulardii*_ *S. boulardii*). Grp 4 “ATB + *S. boulardii*_Vehicle” *n* = 8; Grp 5 “ATB + *S. boulardii S. boulardii*” *n* = 8, the experiment was performed 2 times. **(A)** Experimental design for the administration of the ATB and probiotic over 23 days. **(B,C)** α–Diversity of the bacterial microbiota (16S rRNA) in the fecal microbiota, described by **(B)** observed species and **(C)** Shannon indices. **(D)** β-Diversity. Principal coordinates analysis of Jaccard distances with each sample colored according to the treatment. Groups were compared using Permanova method. **(E,F)** Taxa selected with the differential abundance analysis based on the negative binomial distribution (DESeq2) with padj <0.05 **(E)** decreased or **(F)** increased by the probiotic treatment.

As with the mycobiota, we evaluated the consequences for the bacterial community when *S. boulardii* administration was continued for one (Supplementary Figure S3A) or two (Figure 4A) weeks after the end of ATB treatment. With regards to the microbiota analysis, we did not identify any clear improvement when *S. boulardii* administration was continued for only 1 week (Supplementary Figures S3B–D), except for an additional decrease in the families *Clostridia vadinBB60* and *Tannerellaceae* (Supplementary Figure S3E). Remarkably, when administered for 2 weeks after the end of ATB treatment (23 days in total), *S. boulardii* facilitated an increase in the number of observed species (Figure 4B) and the α-diversity (Shannon index) (Figure 4C) compared with mice that received *S. boulardii* only during ATB treatment. Similar to that after 10 and 16 days, the microbiota were globally different after 23 days and clustered separately in the β-diversity analysis (Jaccard index) (Figure 4D). Again, *S. boulardii* maintained a decrease in the levels of *Clostridia vadinBB60 group* and *Tannerellaceae* families and an increase in the levels of *Lachnospiraceae* and *Oscillospiraceae* (Figures 4E,F). With respect to the levels of the other families with antibiotic-resistant bacteria, *Enterobacteriaceae*, *Enterococcaceae*, *Monoglobaceae*, and *Peptostreptococcaceae*, *S. boulardii* administration only during ATB treatment seemed to be sufficient to maintain the low levels of these bacteria.

### One week after stopping all treatments, *Saccharomyces boulardii* facilitated the restoration of eubiosis

**Figure 5:**
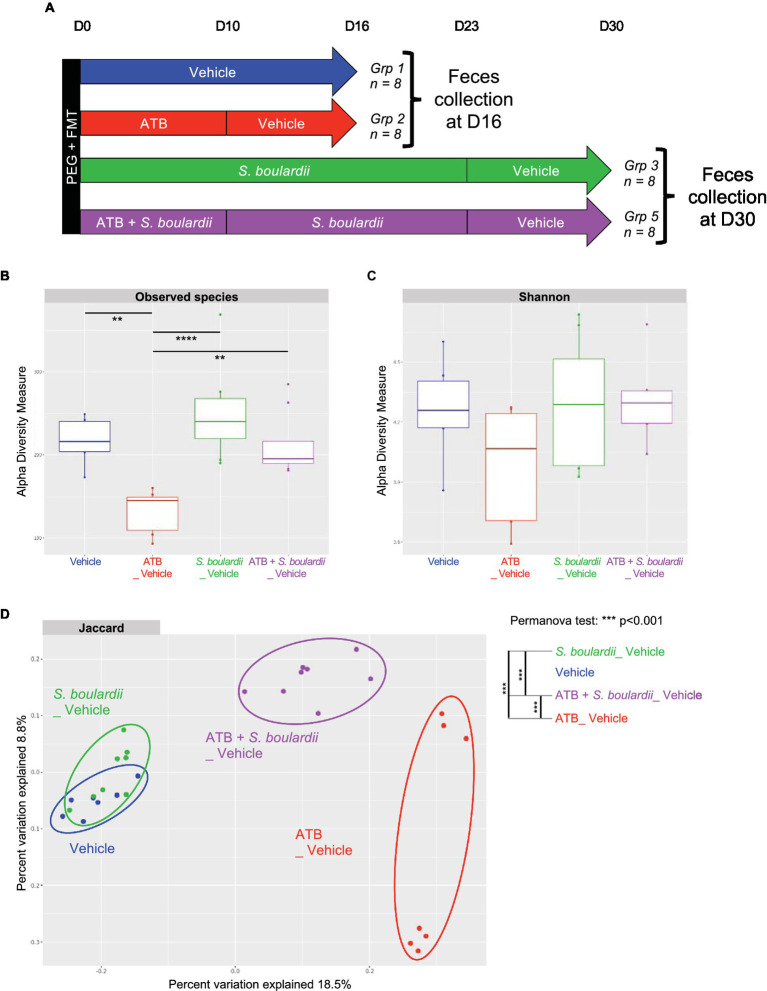
One week after stopping all treatments, *S. boulardii* facilitated the restoration of eubiosis. **(A–D)** HMA mice treated (ATB _ Vehicle) or not (Vehicle) with the ATB for 10 days and *S. boulardii* for 30 days with (ATB + *S. boulardii* _ Vehicle) or without the ATB (*S. boulardii* _ Vehicle). Grp 1 “Vehicle” *n* = 8; Grp 2 “ATB_Vehicle” *n* = 8; Grp 3 “*S. boulardii*_Vehicle” *n* = 8; Grp 5 “ATB + *S. boulardii*_Vehicle” *n* = 8, the experiment was performed 2 times. **(A)** Experimental design for the administration of the ATB and probiotic over 30 days. **(B,C)** α–Diversity of the bacterial microbiota (16S rRNA) in the fecal microbiota, described by **(B)** observed species and **(C)** Shannon indices. **(D)** β-Diversity. Principal coordinates analysis of Jaccard distances with each sample colored according to the treatment. Groups were compared using Permanova method.

Finally, we examined the composition of the bacterial microbiota 1 week after the cessation of all treatments (Figure 5A). As expected, α-diversity, evaluated by observed species or the Shannon index, was reduced by ATB treatment. This reduction was prevented by *S. boulardii* probiotic treatment (Figures 5B,C).

Interestingly, β-diversity analysis showed that the microbiota of HMA mice untreated or treated only with *S. boulardii* 1 week after the end of treatment were very similar to each other (Figure 5D), suggesting that if *S. boulardii* had influenced the bacterial microbiota by itself, this effect did not last. However, these two clusters were very different from the cluster of microbiota exposed to ATB only. Consequently, even after 1 week without the ATB, the bacterial microbiota was still profoundly different from the initial microbiota. Remarkably, if the HMA mice were treated with *S. boulardii* during and after ATB treatment, the newly formed microbiota differed clearly not only from the microbiota only treated with ATB but also from the starting untreated microbiota, suggesting an intermediate state reached with *S. boulardii* administration.

## Discussion

Our objective was to study the effects of supplementation with the probiotic *Saccharomyces boulardii* CNCM I-745 during ATB treatment on both the bacterial and fungal gut microbiota. Indeed, no study has focused on the effect of this probiotic on the mycobiota, whereas it is widely used in human health. It was thus necessary to describe more precisely the impact of its administration on the fungal populations of the gut microbiota.

Our observations were made *in vivo* after FMT with feces from a healthy human donor. The FMT technique transfers a fecal suspension from a healthy donor after intestinal cleansing with polyethylene glycol, according to the published protocol (22). This technique allowed the transfer of part of the human microbiota without using germ-free mice that are immunologically immature or antibiotics, which for obvious reasons could not be used in our study on the effect of ATB (22). Therefore, this technique allowed us to work with a gut microbiota closer to humans than simple conventional mice.

*S. boulardii* CNCM I-745 is widely used in medicine to prevent and reduce the occurrence of diarrhea associated with ATB treatment (14). Its impact on the composition of the bacterial gut microbiota during ATB therapy has been studied in various contexts in humans (13). In the present study, we provide a comprehensive characterization of the change in both fungal and bacterial gut microbiota in human microbiota-associated mice triggered by supplementation with *S. boulardii* during and after ATB therapy with amoxicillin-clavulanate.

The first part of the study investigated the benefit of *S. boulardii* on mycobiota alterations during ATB therapy using fecal ITS rRNA sequencing. ITS rRNA data analysis was hampered by the daily oral gavage with yeast, which biased the prevalence analysis due to an overwhelming quantity of *Saccharomyces* in one group. However, using a differential abundance analysis tool (DESeq2) examining the levels of specific families that were modified, we were able to show that *S. boulardii*, when given during ATB treatment, increased the abundance of the *Tremellaceae* family. The genus *Tremella*, rich in polysaccharides, protein, dietary fiber and vitamin D, has been used for centuries in Chinese medicine to treat chest congestion, asthma and constipation; to balance blood sugar levels and cholesterol; and to reduce inflammation (35). Polysaccharides from this genus have been described as potent anti-inflammatory molecules (36). While the relative abundance decreases of *Atheliaceae*, *Debaryomycetaceae*, *Erysiphaceae*, *Hypocreales* and *Pleosporaceae* families was clear and probably due to the gavage with *S. boulardii*, the absolute quantification would be needed to conclude on the effect on these specific families. Overall, these data suggest that yeast supplementation during ATB treatment do modify the fungal population but there is a need for alternative technics to completely characterize these modifications. One week after the end of all treatments, the composition of the mycobiota was similar between all groups. These results advocate that the probiotic yeast *S. boulardii* CNCM I-745 administration might slightly affect the fungal population but that the effects do not last long after the end of probiotic treatment.

The second part of this study investigated the beneficial effect of *S. boulardii* on alterations in the bacterial microbiota during ATB therapy using fecal 16S rRNA sequencing. Administration of *S. boulardii* can have a slight impact on the bacterial composition of healthy subjects (37). When administered during ATB treatment, *S. boulardii* reduced the levels of the families *Clostridia vadin BB60 group*, *Enterobacteriaceae*, *Enterococcaceae*, *Monoglobaceae*, *Peptostreptococcaceae* and *Tannerellaceae*. These families were indeed favored in the gut after ATB treatment due to their antibiotic resistance to amoxicillin-clavulanate and the release of niches, particularly the *Clostridia vadin BB60 group* and *Enterobacteriaceae* families, given the high levels in mice on ATB without *S. boulardii*. Among the *Enterobacteriaceae* family, *Escherichia coli*, *Klebsiella*, *Salmonella* and *Shigella* species are responsible for diarrhea, fever and emerging antibiotic resistance (38). *Enterococcus faecalis* and *E. faecium* can cause systemic infections and result in urinary tract, intra-abdominal, pelvic and soft tissue infections (39, 40). *Peptostreptococcus* is associated with CRC risk, as its levels are significantly higher in CRC patients than in controls (41). On the other hand, the probiotic yeast was associated with an increase in the levels of *Acidaminococcaceae*, *Akkermansiaceae*, *Atopobiaceae*, *Christensenellaceae*, *Lachnospiraceae*, *Marinifilaceae*, *Muribaculaceae*, *Oscillospiraceae*, *Rikenellaceae*, *Ruminococcaceae* and *Sutterellaceae* families compared to mice that did not receive *S. boulardii* during ATB therapy. The probiotic *Akkermansia muciniphila* has been reported to reduce the risks of diabetes and obesity (42). The abundance of the *Christensenellaceae* family, inversely related to host body mass index and thus metabolic health, has been associated with human longevity and may also play a role in obesity and IBD (43). Additionally, with regard to obesity, *Muribaculaceae* appeared to be overrepresented in a group of mice fed a high-fat diet (44). The *Lachnospiraceae* family has been widely studied due to its ability to produce short-chain fatty acids and has therefore been associated with reduced severity of several diseases, such as IBD and CRC. *Blautia hansenii* in particular has been identified as protective in a mouse model of IBD (41, 45). The genus *Alistipes*, belonging to the family *Rikenellaceae*, is decreased in those with liver microbiota-related diseases and IBD and thus may play a protective role in the progression of cirrhosis and liver fibrosis, as well as colitis and CRC (46). Among the *Ruminococcaceae* family, *Faecalibacterium prausnitzii* has been particularly studied in regards to IBD and has shown anti-inflammatory properties *in vitro* and *in vivo* (47, 48).

Taken together, these data suggest that the administration of *S. boulardii* CNCM I-745 during ATB treatment helps preserve the microbiota from pathogenic bacteria and promotes certain bacteria identified as beneficial to host health. When supplementation was continued for one to 2 weeks after the end of ATB treatment, the yeast continued to maintain a low level of antibiotic-resistant bacteria, almost undetectable for the *Enterobacteriaceae* family, and promote higher levels of the family *Lachnospiraceae,* which is considered beneficial to host health. Finally, one week after stopping all treatments, mice that received *S. boulardii* during and after ATB therapy showed an intermediate state, promoting a return to eubiosis for the bacterial microbiota. These results suggest that *S. boulardii* should be given during ATB therapy instead of immediately after and should be continued after the end of therapy for at least 2 weeks. However, it is important to keep in mind that these results have been obtained with mice groups of limited size (8 to 12) for each time point and some other variations can have been missed or some over-evaluated.

In conclusion, it clearly appears that the administration of *S. boulardii* CNCM I-745 during amoxicillin-clavulanate treatment has a deep impact on microbial equilibrium. The identification of the microorganisms modulated by the yeast probiotic suggests that its use during ATB treatment is beneficial in many aspects regarding the bacterial microbiota, mostly by promoting beneficial microorganisms and keeping the development of deleterious ones at a minimum. In addition, prolonging the treatment at the end of ATB therapy extends these beneficial effects on the bacterial community.

## Data availability statement

The datasets presented in this study can be found in online repositories. The names of the repository/repositories and accession number(s) can be found at: https://www.ncbi.nlm.nih.gov/, PRJNA896895.

## Ethics statement

The animal study was reviewed and approved by COMETHEA C2EA – 45, Jouy en Josas, France.

## Author contributions

MS and MR designed the study and analyzed and interpreted the data. MS, YW, AL, GC, CM, and CD helped to collect the data. MS and MR wrote the manuscript. M-LM, PL, and HS commented on and corrected the manuscript. All authors contributed to the article and approved the submitted version.

## Funding

The authors declare that this study received funding from Biocodex. The funder was not involved in the collection, analysis and interpretation of data, nor the writing of this article. The original study design was initially discussed between the research group and the funder. The decision to submit the data for publication was approved by the funder.

## Conflict of interest

The authors declare that the research was conducted in the absence of any commercial or financial relationships that could be construed as a potential conflict of interest. The original study design was initially discussed between the research group and Biocodex. The decision to submit the data for publication was approved by Biocodex.

## Publisher’s note

All claims expressed in this article are solely those of the authors and do not necessarily represent those of their affiliated organizations, or those of the publisher, the editors and the reviewers. Any product that may be evaluated in this article, or claim that may be made by its manufacturer, is not guaranteed or endorsed by the publisher.

## Footnotes

^1^http://frogs.toulouse.inra.fr/

## Abbreviations

ANOVA: analysis of variance
ATB: antibiotic
CRC: colorectal cancer
FMT: fecal microbiota transfer
Grp: group
HMA: human microbiota-associated
IBD: inflammatory bowel disease
IBS: irritable bowel syndrome
LDA: linear discriminant analysis
NaCl: sodium chloride
NGS: next-generation sequencing
PCoA: principal coordinates analysis
PEG: polyethylene glycol
*S. boulardii*: *Saccharomyces boulardii*
SEM: standard error of the mean
