# Supplementary figures and images for: Saccharomyces boulardii CNCM I-745 supplementation during and after antibiotic treatment positively influences the bacterial gut microbiota
Source: Front Med (Lausanne). 2023 Aug 4;10:1087715. doi: 10.3389/fmed.2023.1087715 (PMC10436532; doi:10.3389/fmed.2023.1087715)

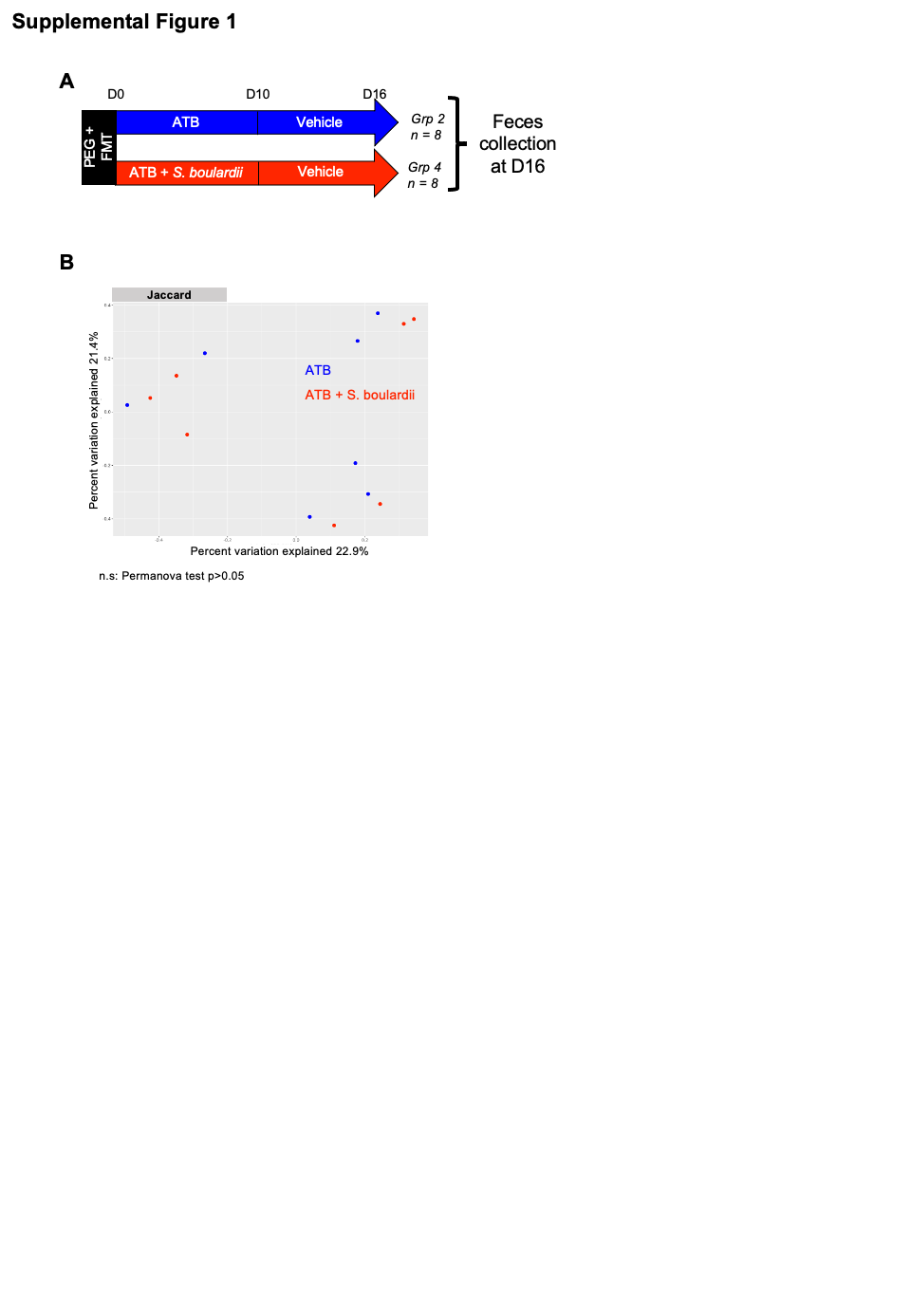

Supplement: Supplementary file 1 [file Image_1.tiff]

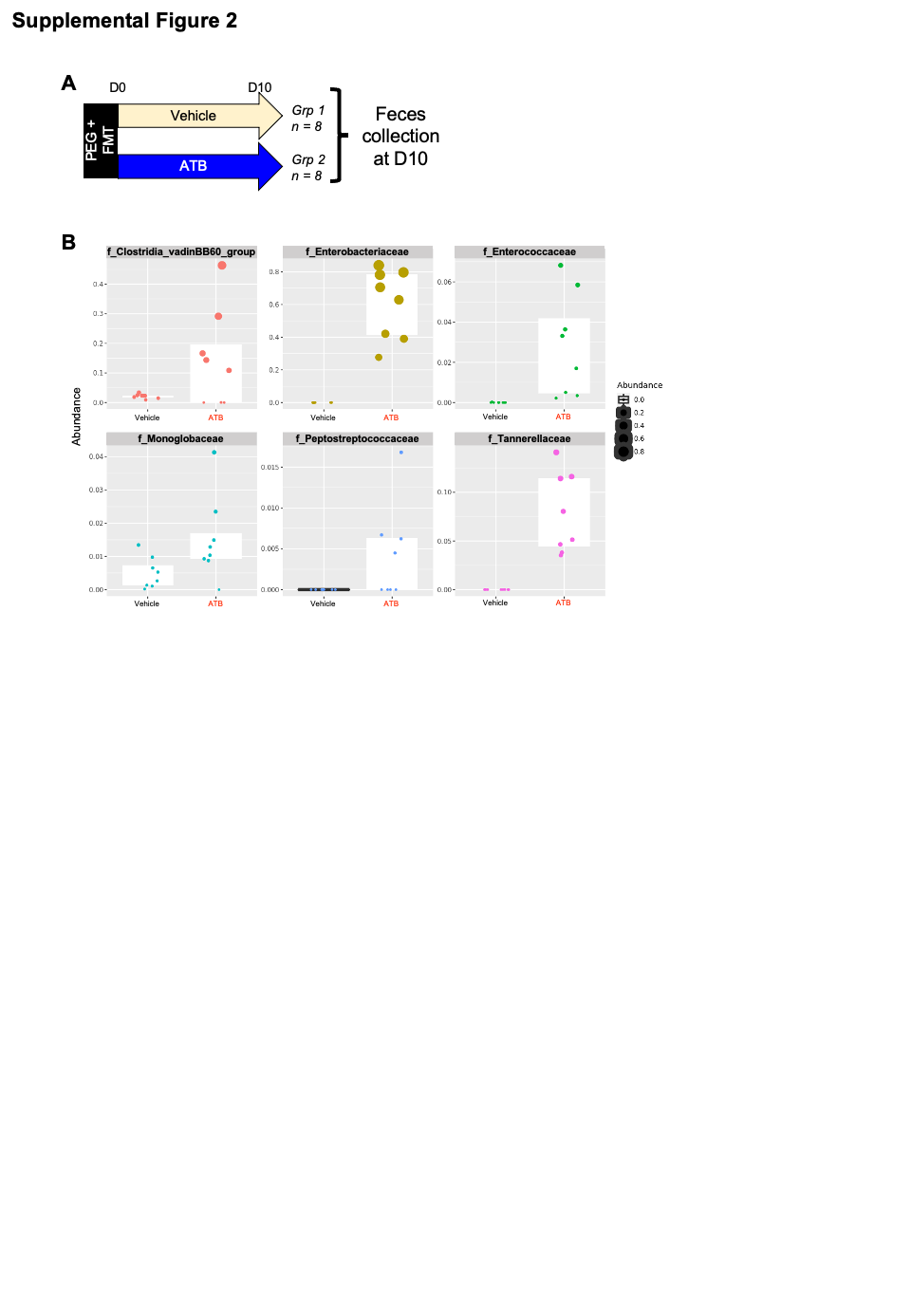

Supplement: Supplementary file 2 [file Image_2.tiff]

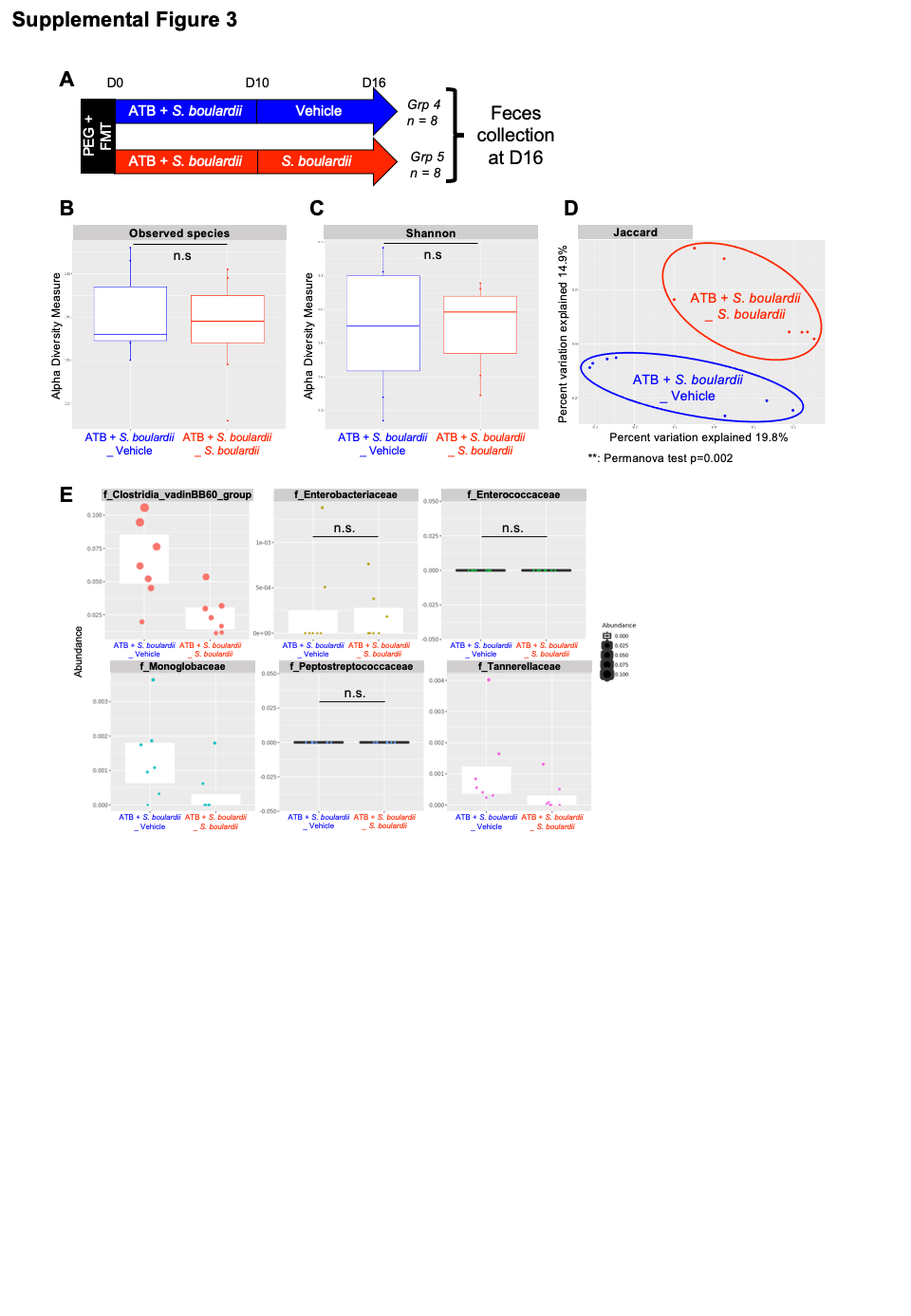

Supplement: Supplementary file 3 [file Image_3.tiff]
